# Placebo Effect of Caffeine on Physiological Parameters and Physical Performance

**DOI:** 10.3390/nu16101405

**Published:** 2024-05-07

**Authors:** David Ortiz-Sánchez, Alfredo Bravo-Sánchez, María Ramírez-delaCruz, Pablo Abián, Javier Abián-Vicén

**Affiliations:** 1 Performance and Sport Rehabilitation Laboratory (DEPORSALUD), Faculty of Sports Sciences, University of Castilla-La Mancha, Avenida Carlos III s/n, 45071 Toledo, Spain; david.ortiz@uclm.es (D.O.-S.); alfredo.bravo@ufv.es (A.B.-S.); maria.ramirez@uclm.es (M.R.-d.); pabian@comillas.edu (P.A.); 2 Faculty of Health Sciences, Universidad Francisco de Vitoria, 28223 Pozuelo de Alarcón, Spain; 3 Faculty of Humanities and Social Sciences, Comillas Pontifical University, Calle. Alberto Aguilera 23, 28049 Madrid, Spain

**Keywords:** ergogenic aids, strength, heart rate variability, expectations, side effects

## Abstract

This study aimed to analyse the placebo effect associated with a high dose of caffeine (9 mg/kg) on heart rate and its variability and on strength tests. Methods: 18 participants experienced in strength training (19.7 ± 2.3 years; 72.2 ± 15.0 kg; 169.6 ± 9.0 cm) performed two days of trials (caffeine-informed/placebo-ingested (placebo) and non-ingested (control)). Firstly, heart rate and its variability were measured while participants lay down for 15 min. After that, bench press and squat tests were performed at 3 different loads (50%, 75% and 90% of 1RM). Perception of performance, effort and side effects were also evaluated. Results: no differences were found in the vast majority of strength variables analysed. Resting heart rate decreased in the placebo trial (60.39 ± 10.18 bpm control vs. 57.56 ± 9.50 bpm placebo, *p* = 0.040), and mean RR increased (1020.1 ± 172.9 ms control vs. 1071.5 ± 185.7 ms placebo, *p* = 0.032). Heart rate variability and perception of performance and effort were similar between conditions (*p* > 0.05 in all cases). Side effects such as activeness and nervousness were reported while consuming the placebo. Conclusions: the placebo effect did not modify performance in the majority of the strength test variables, HRV and perception of performance and effort. However, resting heart rate was reduced, mean RR increased, and some side effects appeared in the placebo trial.

## 1. Introduction

The use of ergogenic aids aimed at athletic performance improvements has increased among young athletes [1]. Caffeine (1, 3, 7-trymethylxantine) is commonly used as an ergogenic aid due its clear benefits for athletic performance [2,3]. These effects have been studied in different activities (i.e., endurance exercise [4], peak and mean power output in anaerobic-based exercise [5] and in maximal strength and bar velocity in strength/power activities [6] in both individual and team sports [7]). The ergogenic effect of caffeine has been widely studied on variables associated with resistance training (i.e., mean propulsive velocity, power and force). In addition, different caffeine doses have been evaluated in both upper and lower body exercises (bench press and squat) at different loads [8,9]. However, only a few studies have analysed both bench press and squat in the same investigation and controversial results were found. Pallares et al. [8] found that a low caffeine dose (3 mg/kg) was enough to improve mean velocity at low loads for bench press and squat exercises, but a higher caffeine dose (9 mg/kg) was needed to improve both exercises at a high load (90% of the one-repetition maximum (1RM)). In contrast, Ruiz-Fernández et al. [9] found that the caffeine ergogenic effect was more pronounced at high loads (≥75% 1RM) in the same exercises with a dose of 3 mg/kg.

The ergogenic effect of caffeine could appear when consumed in doses of 3–6 mg/kg body mass [10,11]. Moreover, it is suggested that 60 min pre-exercise could be the optimal timing due the plasma concentration peak [12]. Despite the safety characteristics of caffeine, high doses (9 mg/kg) can produce several side effects (i.e., tachycardia, nervousness, gastrointestinal discomfort and insomnia) appearing immediately or even 24 h after intake [13]. It has been shown that higher caffeine doses increase the magnitude and prevalence of these side effects [13]. Therefore, it would be interesting to find a strategy that maintains the ergogenic benefits of caffeine but eliminates the side effects. Some authors have found that the belief in caffeine ingestion (while no caffeine is consumed) may improve performance. Hence, this suggests that the placebo effect can be used to improve athletes’ performance with a reduction in the possible adverse side effects of the supplement (e.g., caffeine) [14].

The placebo effect, defined as a desirable outcome resulting from a person’s expected and/or learned response to a treatment or situation [15], has generated great interest in sports science research. A placebo is usually used as a control treatment, theoretically indistinguishable from the experimental treatment, but it does not have the biological or mechanical active component [16]. Making athletes believe they are taking a performance-enhancing supplement (e.g., caffeine) may improve performance in resistance exercises. It has been shown that a caffeine placebo enhanced performance in bench press [17] and single-leg extension [18,19] tests and benefited substrate oxidation during exercise [20]. Conversely, some authors have observed that belief in caffeine ingestion (caffeine-informed/placebo ingested) is not enough to improve maximal voluntary concentric force and strength endurance [21,22]. Thus, the existing literature on the placebo effect of caffeine in resistance exercise is limited and controversial, and more research is needed on this topic. This is not only the case for resistance exercise, since the placebo effect of caffeine has also not been extensively studied regarding physiological parameters such as heart rate variability (HRV).

The autonomic nervous system regulates cardiovascular function and induces changes in heart rate (HR) via inhibition of the parasympathetic tone or stimulation of the sympathetic tone. Both the sympathetic and parasympathetic tones are present in increasing HR during exercise and during recovery after exercise [23]. The physiological functioning of the autonomic nervous system can be studied via HRV, since it analyses the peak-R to peak-R intervals of consecutive cardiac beats [24]. It has been shown that caffeine in moderate doses may increase HRV values such as RMSSD [25]. However, the latest systematic review on the effect of caffeine intake on HRV showed that most of the studies analysed found no changes with doses of 1–5 mg/kg [26]. Moreover, to our knowledge, there is only one investigation that has analysed the belief in caffeine intake on HRV, and it showed no changes in HRV in healthy adults [27]. Hence, the placebo effect of caffeine on HRV should be further investigated.

Therefore, the main objective of this study was to analyse the placebo effect associated with the belief in the intake of a high dose of caffeine (9 mg/kg) on HRV, resting HR and both upper and lower body strength tests.

## 2. Materials and Methods

### 2.1. Study Design

A repeated, randomized and counterbalanced experimental design was used to compare the effects of ingestion of a placebo presented as caffeine (placebo) and a control situation where no substance was ingested (control) on physiological variables at rest and on strength test performance. All participants underwent an initial familiarisation session. Subsequently, they underwent two days of testing in which, on one day, they took 9 mg/kg of cellulose (placebo), thinking it was caffeine, while the other day they took nothing. The order of on which day they took the placebo was randomly determined in a counter-balanced way.

### 2.2. Participants

An a priori sample size estimation was calculated based on the effect size obtained with placebo vs. control conditions in Campelo et al.’s investigation [17]. This calculation was performed with Suresh and Chandrashekara’s formula [28]. The sample size estimation for attaining a power of 0.9 and a bilateral alpha level of 0.05 revealed that at least 12 participants were required. Eighteen physically active people (19.7 ± 2.3 years; 72.2 ± 15.0 kg; 169.6 ± 9.0 cm), of whom 12 were men and 6 women, voluntarily participated in the study. The inclusion/exclusion criteria were that they had to be aged between 18 and 35 years old, free from any kind of injury, with a minimum of 6 months’ resistance training experience, and that they did not consume other sport supplements during the trial [9]. Participants had previous experience with caffeine supplementation, and all of them were categorized as moderate consumers (3–6 mg/kg/day) according to their habitual caffeine consumption as per previous proposed thresholds for classifying individuals in sport performance research [29].

### 2.3. Procedures

First, a familiarisation session was held in which participants were informed about the study and had the opportunity to ask questions to clarify any possible doubts. The participants were also informed of the benefits of caffeine for physical performance and its possible side effects. They were also told to refrain from caffeine and strenuous exercise 24 h before each visit [9]. After the familiarisation session, the study comprised two more sessions during which evaluations were made. On one day, the participants were informed they were taking a high dose of caffeine (9 mg/kg), but they were actually taking cellulose (placebo) at the same dose (9 mg/kg), while on the other day, they did not ingest any capsules (control). Participants were told that this was a control situation to assess the effect of caffeine on strength test performance. Placebo was consumed in a capsule so participants could not identify the taste of the substance. The order that established on which day they would take the placebo was determined randomly.

During the familiarisation, participants were measured and weighed with a calibrated scale (Seca 769, Seca GMBH, Hamburg, Germany). After that, they practised the tests to be carried out during the study and they were able to ask any questions they had about them. A test was also carried out to determine the 1RM of each participant in back squat and bench press, so that the load used during the two days of testing would be individualised and always the same. Bench press and back squat exercises were selected as they represent major upper- and lower-body muscle group loads and both have been studied previously to analyse the ergogenic effect of caffeine on muscular strength, power and endurance [30]. Both exercises were performed using a Smith machine (Multipower, Technogym, Spain) in which 2 vertical guides regulated the barbell movement. Once the familiarisation was completed, participants attended two days of testing with at least 48 h between both days [10]. For both testing days the protocol was carried out in the same conditions and at the same time of the day (between 9:00 a.m. and 11:30 a.m.).

On the day they had to take the placebo, they were called to the laboratory 60 min before the start of the tests in order to consume the capsule. Hence, when they started the tests would coincide with the time it takes for the caffeine to take effect. During these 60 min, the participants did not engage in any physical activity. On the day they did not have to take a supplement, they went straight to the lab to carry out the tests. The protocol for both days of testing was the same. The first test was a measurement of HRV and resting HR. Once this first test was completed, the participants performed the strength tests, first the bench press test and then the squat test.

After each session, participants were asked to answer questions about their perceived exertion during the strength tests. In addition, they were sent a side effect questionnaire to fill in 24 h after taking the supplement.

### 2.4. Analysis of Resting Heart Rate and Heart Rate Variability

Participants were positioned supine on a treatment bed for 15 min with a Polar H10 chest strap HR monitor (Polar Electro Oy, Kempele, Finland). The participants relaxed for 10 min before the data were taken and 5 min after the data were taken [31].

The variables measured were resting HR, the average time between RR intervals (mean RR), which is inversely proportional to HR, and four HRV variables. Linear methods in time were applied to analyse HRV. In the time domain indexes, the variables analysed were the square root of the average of the square of the differences between normal adjacent RR intervals (RMSSD) and standard deviation of the average of all normal RR intervals (SDNN). The Poincaré plot indexes were of the standard deviation of the instantaneous rate variability. The rhythm (SD1) and standard deviation of long-term continuous RR interval variability (SD2) were also analysed [32].

Kubios HRV Premium analysis software version 4.1.0 (Kubios, Biosignal Analysis and Medical Image Group Department of Physics, University of Kuopio, Finland) [31] was used to analyse all the variables.

### 2.5. Strength Tests

During the familiarisation session, the 1RM test of both exercises, back squat and bench press, was determined for each participant following a previously described protocol [8]. Participants started performing 3 repetitions with a low load (only the bar, 12 kg), which was increased by 10–15 kg until mean velocity reached 0.5 m/s in the bench press and 0.8 m/s in the squat, and then started to perform only one repetition. A rotatory encoder (Isocontrol, Madrid, Spain) was used to measure the velocity. After that, the load was adjusted with smaller increments (<5 kg) to precisely determine 1RM. The heaviest load that the participant was able to properly lift was considered their 1RM.

Participants performed a standardised warm-up protocol on both testing days. It included pedalling on a cycle ergometer and a submaximal attempt on the first exercise that was about to be carried out, in this case the bench press. Once this first exercise was completed, participants performed a new submaximal attempt for the squat before starting with the squat test [33].

For both the bench press and squat tests the same protocol was used [9]. Each exercise used 3 loads (50%, 75% and 90% of 1RM). The participants performed 2 repetitions at 50%, 1 repetition at 75% and 1 repetition at 90%, with a passive rest of 3 min between loads. Each repetition was performed at the maximum possible speed, and the repetition of each load that was performed at the highest speed was used for the evaluation. In both tests, concentric form was analysed in isolation. For this purpose, two supports were placed where the bar could be supported at the lowest point of the exercise. In the bench press test, it was the point where the bar was slightly above the chest and for the back squat, it was the point where the participant was parallel to the floor. The bar holders were used to rest the bar for 2 s before the participants performed the concentric phase on the researcher’s signal. All repetitions were measured using a rotatory encoder (Isocontrol, Spain) in order to measure mean propulsive velocity (Vmean), peak velocity (Vpeak), mean acceleration (Amean), peak acceleration (Apeak), mean power (Wmean), peak power (Wpeak), mean force (Fmean), peak force (Fpeak) and rate of force development (RFD).

At the end of each session, participants were required to fill out a questionnaire about their perception of power, endurance, exertion (RPE), muscle soreness [9] and perception of performance [34]. This questionnaire included a scale from 1 to 10 points to assess each item.

### 2.6. Side Effects Evaluation

Twenty-four hours after the evaluation session, participants responded to an online survey about discomforts typically associated with caffeine ingestion. This survey included 9 items on a yes/no scale. This questionnaire was based on previous publications about side effects derived from the ingestion of caffeine [13,35]. It included nervousness, insomnia, gastrointestinal problems, activeness, irritability, muscle pain, headache, tachycardia/increased heart rate and increased urine production.

### 2.7. Statistical Analysis

Results of quantitative data are shown as mean ± standard deviation. The normality of the variables was checked with the Shapiro–Wilk test. Once the normality of the variables was assumed (*p* > 0.05), a paired *t*-test was used to analyse each of the variables. Effect size was calculated (Cohen’s d) for each variable. Results of qualitative data (side effects) are presented as percentages. Differences in side effects were analysed using the McNemar test.

The level of significance was set at *p* < 0.05 in all cases. All calculations were performed with IBM SPSS Statistics 28.0 (SPSS, Chicago, IL, USA).

## 3. Results

### 3.1. Strength Test

Mean velocity (Vmean) was significantly higher (*p* = 0.040) by 2.4 ± 5.0% in the control versus placebo condition during the bench press at a load of 50% of 1RM. No more differences were found in any of the variables analysed (*p* > 0.063). Table 1 shows the bench press test results at 50%, 75% and 90% loads.

Table 2 shows the same results for the back squat test at 50%, 75% and 90% loads. RFD increased significantly by 10.0 ± 16.7% in the control trial during the squat test at a load of 75% of 1RM. No further differences were found in the rest of the variables analysed (*p* > 0.065). In addition, two participants were not able to complete the last repetition (90% of 1RM) in either session (control and placebo).

### 3.2. Analysis of Resting Heart Rate and Heart Rate Variability

Both HR (4.3 ± 8.9%) and mean RR (5.4 ± 9.1%) were significantly decreased in the placebo trial (*p* < 0.05). No differences appeared in the rest of the variables (*p* > 0.900). Table 3 shows the results of the HRV and resting HR variables.

### 3.3. Perception Questionnaire

No differences were found in any of the variables analysed in the perception questionnaire, as the results of the 1 to 10 points scale show for power (7.44 ± 1.70 vs. 8.00 ± 1.64 points, *p* = 0.056), endurance (7.36 ± 1.53 vs. 7.97 ± 1.27 points, *p* = 0.152), exertion (6.44 ± 2.10 vs. 6.42 ± 2.30 points, *p* = 0.932), muscle soreness (2.89 ± 2.47 vs. 2.28 ± 1.93 points, *p* = 0.127) and performance (7.25 ± 1.42 vs. 7.42 ± 1.55 points, *p* = 0.589).

### 3.4. Side Effect Evaluation

Table 4 shows the side effect evaluation of all 9 items, expressed as a percentage, if participants had any of them during the 24 h after taking the placebo. Significant differences appeared in nervousness, activeness, tachycardia and increased urine production (*p* < 0.05).

## 4. Discussion

Caffeine has been widely used due to the benefits it has for athletic performance [2,3]. However, due to its possible side effects, especially when consumed in high doses, it seems to be an interesting strategy to make the participants believe they are taking caffeine while they are consuming a placebo [14]. The main objective of this study was to analyse the placebo effect associated with the belief in the intake of a high dose of caffeine (9 mg/kg) on HRV, resting HR and both upper- and lower-body strength tests. Our main findings were that significant changes were revealed in the mean velocity of the bench press and in the RFD in the squat. Regarding the assessment of HRV and resting HR, we found a significant decrease in the resting HR and an increase in the mean RR. Moreover, the prevalence of side effects was significant in nervousness, activeness, tachycardia and increased urine production, while no differences were found in the rest of the variables. Finally, in the perception of performance and effort, we found no significant differences between the control condition and the placebo condition. Therefore, the belief in caffeine intake could improve some aspects of strength test variables and resting HR, but more research is needed regarding the placebo effect on other variables that may improve physical performance.

It has been found that caffeine improves performance in resistance exercises (i.e., bench press and squat) [8,9]. However, there is controversy over the placebo effect of caffeine in resistance exercises, and there is little research on the subject. In this study, we observed a significant increase in two variables of the strength tests (mean velocity bench press at 50% of 1 RM, *p* = 0.040; and RFD squat at 75% of 1RM, *p* = 0.031) in the placebo trial versus the control trial. However, we found no significant differences in the other strength variables studied. Our results are in agreement with Tallis et al. [21], who found that belief in caffeine intake did not enhance performance on measures of maximal voluntary strength (i.e., maximal peak and average force and the ability to maintain peak and average force, after 40 repeated contractions of both knee extensors and flexors) and Filip-Stachnik et al. [22], who found no placebo effect of caffeine in muscle strength and strength endurance during the bench press exercise in women. In contrast, other authors found that subjects’ belief that they had taken caffeine enhanced the number of repetitions in a bench press test to failure at 80% of 1RM [17] and in a single-leg extension test to failure at 60% of 1RM [18,19]. Hence, despite the little literature on this topic, it seems that the placebo effect of caffeine may be dependent on the outcome variables being assessed. Caffeine may be used as an effective ergogenic aid, but it is not clear that just the expectancy of taking this supplement will enhance resistance performance [21]. Thus, the existing literature is controversial, and more research is needed on the placebo effect in resistance exercises.

Some controversy has been found regarding the effect of caffeine intake on HR and HRV [26]. Moreover, to our knowledge there has only been one investigation regarding the placebo effect of caffeine on HRV, and it showed no changes [27]. We found a significant decrease (*p* = 0.040) in resting HR when participants believed they had taken caffeine. Our outcome is contrary to other results, which were that HR was not affected by the placebo effect [27], although some authors have found that caffeine intake (5 mg/kg) reduced resting HR [36]. Hence, HR was affected by the belief in caffeine intake in the same way as in this investigation with caffeine intake. Furthermore, the mean RR increased (*p* = 0.032) in the placebo trial versus control trial. Regarding HRV, it has been observed that caffeine can increase HRV variables such as RMSSD [25]. However, our results showed that taking a placebo, believing it was caffeine, did not generate changes in HRV variables. The results of our study are similar to those of Domotor et al. [27], which to date is the only research that has studied the placebo effect of caffeine on HRV in healthy adults. Therefore, HRV can be affected by caffeine, but it is not enough to believe that caffeine has been consumed (i.e., placebo intake). Nevertheless, more research is needed to obtain conclusive results.

We evaluated the perception of performance and effort (RPE) in both control and placebo trials. The results showed no differences in any of the variables (i.e., power, endurance, muscle soreness, RPE and overall performance). Similar to our results, some authors that evaluated the RPE in resistance exercises found that the rate of perceived muscle fatigue was not modified with the placebo effect of high doses [19]. In contrast, other studies reported that the RPE in activities such as the bench press and single-leg extension to failure was lower when participants believed they had consumed caffeine in moderate doses (~3 mg/kg) [17,18]. In other activities such as running, the placebo effect and its RPE have also been studied. It has been shown that in maximal efforts, RPE did not change between the placebo and control condition, even though there was an improvement in performance in the placebo condition [14]. Most of the participants reported that they did not notice any enhancement effect during the tests in comparison with their previous experience with caffeine supplementation. Therefore, it seems that the expectations they had about the effect of the caffeine they had taken were too high and did not meet the reality. As a consequence, performance could have been affected, which led to some results that may seem odd (i.e., decrease of HR or decrease in performance while thinking they are consuming caffeine). Hence, we may conclude that a high dose of placebo may present opposite effects in participants with previous experience in acute caffeine intake.

Finally, we evaluated the following side effects proposed by de Souza et al. [13] and Salinero et al. [35] in their studies: nervousness, insomnia, gastrointestinal problems, activeness, irritability, muscle pain, headache, tachycardia/increased heart rate and increased urine production. Our results indicated that the placebo increased nervousness, activeness, tachycardia and urine production, with activeness being the most reported side effect. This would be in agreement with the literature, since similar investigations showed an increase in activeness after the placebo intake [14]. These side effects commonly appear after caffeine intake [13,35]. However, our participants did not consume caffeine but only a placebo. The side effects of high doses of caffeine are well known [13]. This may be one of the reasons that made participants perceive that they were having those side effects due to a high caffeine dose intake. Consequently, it seems that the blinding of the study was effective, since participants thought they had consumed caffeine. Nevertheless, this outcome is not a practical one, since side effects appeared, but performance was not enhanced. It may be interesting to use another type of ergogenic aid whose side effects subjects are unaware of.

As practical applications, coaches and physical trainers of sports whose main physical activities are those related to the strength tests analysed in this study (bench press and squat) must take into account that making athletes think that they are taking a higher dose of caffeine than they usually do is not effective to improve their performance, nor does it modify the perception of performance of effort. Moreover, despite not consuming caffeine, the belief in consuming a high dose of caffeine produces side effects associated with caffeine intake. Finally, coaches should consider the previous experience of their athletes with caffeine supplementation and that the placebo effect seems to be dependent on the variables analysed.

Our study is not free of limitations. Participants had previous experience with resistance training, but they were recreationally trained, so these results should be considered for this kind of population. Future research should be conducted with different populations in order to better comprehend the placebo effect. Moreover, something that should be taken into account is the previous experience of participants with caffeine supplementation. It appears that with participants that have experienced the effect of acute caffeine intake, it is not effective to make them believe that they are consuming a higher dose of caffeine than usual, since the effect they are expecting is even higher than the one they are used to. Therefore, it would be interesting to analyse participants with different experience in training, different caffeine intake experience (regular vs. not regular consumers) and different doses of caffeine intake experience. It has been reported that the placebo effects induced by expectation are smaller compared to pre-conditioning situations [16]. Despite both techniques being used, as participants knew of the caffeine effect beforehand, it seems that expectation played a vital role. In this investigation, participants were told that they were about to consume a higher dose of caffeine than their usual one, making it possible that their expectations of the effect they were going to experience were really high. Even though other authors found that making the participants believe they had consumed 9 mg/kg of caffeine improved their performance more than making them believe they had consumed a placebo or 4–5 mg/kg of caffeine [37], it seems it is the contrary in this investigation. Some of the participants even reported that they were having second thoughts about the idea of consuming that amount of caffeine. Hence, it may have affected their performance during the tests. Additionally, some variables are close to present statistical significance (i.e., W mean at 50% of 1-RM and V peak and A mean at 75% of 1-RM for the bench press test). Therefore, despite calculating the a priori sample size required for this study and recruiting more participants than the minimum necessary, it is possible that with a bigger sample size more statistical significance may appear. Finally, aspects such as nutrition or the phase of the menstrual cycle were not considered. Future research could collect biological samples in order to confirm the absence of caffeine and analyse the phase of the menstrual cycle.

## 5. Conclusions

In conclusion, the placebo effect of a high dose of caffeine (9 mg/kg) did not modify performance regarding the vast majority of variables analysed in the bench press and squat tests at different loads. Additionally, resting HR was significantly decreased by the placebo effect, but HRV was not modified. Although caffeine was not actually consumed, participants reported a significant appearance of nervousness, activeness, tachycardia and increased urine production. Finally, perception of performance and effort did not change with the placebo effect of caffeine. Therefore, a high dose of placebo in participants with previous experience in acute caffeine intake does not present the expected effects and in some cases presents the opposite effect.

## 6. Key Points

- The placebo effect of a high dose of caffeine does not enhance the vast majority of strength variables.
- The placebo effect of a high dose of caffeine reduces resting heart rate but does not modify heart rate variability.
- The placebo effect of a high dose of caffeine does not change the perception of performance or effort during resistance exercise.
- The belief in consuming a high dose of caffeine causes side effects associated with caffeine consumption.
- The expectation of consuming a high dose of caffeine is not beneficial for performance enhancement.

## Figures and Tables

**Table 1:** Difference between control trial (CON) and placebo trial (PLA) for the bench press tests at 50%, 75% and 90% of 1RM.

VARIABLE	CON	PLA	*p*	Cohen’s d	CI 95%
50% 1RM					
Vmean (m/s)	0.78 ± 0.07 *	0.76 ± 0.07	0.040 *	0.5	0.1 to 1.0
Vpeak (m/s)	1.28 ± 0.14	1.25 ± 0.15	0.101	0.4	−0.1 to 0.9
Amean (m/s^2^)	3.86 ± 0.59	3.70 ± 0.55	0.110	0.4	−0.1 to 0.9
Apeak (m/s^2^)	13.28 ± 3.48	12.66 ± 3.56	0.187	0.3	−0.1 to 0.8
Wmean (W)	272.9 ± 98.9	265.5 ± 98.3	0.063	0.5	−0.0 to 0.9
Wpeak (W)	519.1 ± 190.5	500.5 ± 185.5	0.127	0.4	−0.1 to 0.8
Fmean (N)	356.5 ± 122.3	354.4 ± 122.1	0.192	0.3	−0.2 to 0.8
Fpeak (N)	534.8 ± 187.2	534.5 ± 205.5	0.983	0.0	−0.5 to 0.5
RFD (N/s)	28,622.2 ± 17,616.5	27,368.7 ± 18,423.0	0.559	0.1	−0.3 to 0.6
75% 1RM					
Vmean (m/s)	0.51 ± 0.09	0.50 ± 0.06	0.381	0.2	−0.3 to 0.7
Vpeak (m/s)	0.85 ± 0.13	0.82 ± 0.12	0.097	0.4	−0.1 to 0.9
Amean (m/s^2^)	1.79 ± 0.46	1.67 ± 0.34	0.081	0.4	−0.1 to 0.9
Apeak (m/s^2^)	8.65 ± 1.52	8.12 ± 1.30	0.110	0.4	−0.1 to 0.9
Wmean (W)	249.4 ± 83.9	246.5 ± 81.6	0.533	0.1	−0.3 to 0.6
Wpeak (W)	472.7 ± 161.5	452.4 ± 146.8	0.133	0.4	−0.1 to 0.8
Fmean (N)	502.2 ± 166.4	502.5 ± 166.8	0.682	−0.1	−0.6 to 0.4
Fpeak (N)	718.9 ± 228.8	729.3 ± 250.6	0.648	−0.1	−0.6 to 0.4
RFD (N/s)	34,119.4 ± 11,382.0	32,453.3 ± 14,532.8	0.534	0.1	−0.3 to 0.6
90% 1RM					
Vmean (m/s)	0.33 ± 0.07	0.32 ± 0.07	0.354	0.2	−0.3 to 0.7
Vpeak (m/s)	0.60 ± 0.11	0.60 ± 0.13	0.847	0.1	−0.4 to 0.5
Amean (m/s^2^)	0.94 ± 0.25	0.89 ± 0.23	0.345	0.2	−0.3 to 0.7
Apeak (m/s^2^)	5.89 ± 1.15	6.09 ± 1.40	0.578	−0.1	−0.6 to 0.3
Wmean (W)	186.9 ± 65.1	184.0 ± 72.2	0.650	0.1	−0.4 to 0.6
Wpeak (W)	385.2 ± 124.8	385.5 ± 136.0	0.984	0.0	−0.5 to 0.5
Fmean (N)	596.4 ± 209.0	595.7 ± 208.1	0.289	0.3	−0.2 to 0.8
Fpeak (N)	841.8 ± 312.2	818.3 ± 313.8	0.434	0.2	−0.3 to 0.7
RFD (N/s)	39,070.3 ± 15,032.4	33,511.0 ± 13,348.6	0.069	0.5	0.0 to 1.0

Vmean = mean velocity (m/s); Vpeak = peak velocity (m/s); Amean = mean acceleration (m/s^2^); Apeak = peak acceleration (m/s^2^); Wmean = mean power (W); Wpeak = peak power (W); Fmean = mean force (N); Fpeak = peak force (N); RFD = rate of force development (N/s); * denotes significant differences between the control trial and the placebo trial (*p* < 0.05).

**Table 2:** Difference between control trial (CON) and placebo trial (PLA) for the squat tests at 50%, 75% and 90% of 1RM.

VARIABLE	CON	PLA	*p*	Cohen’s d	CI 95%
50% 1RM					
Vmean (m/s)	0.76 ± 0.06	0.75 ± 0.06	0.375	0.2	−0.3 to 0.7
Vpeak (m/s)	1.41 ± 0.14	1.41 ± 0.10	0.971	0.0	−0.4 to 0.5
Amean (m/s^2^)	4.18 ± 0.63	4.04 ± 0.67	0.229	0.3	−0.2 to 0.8
Apeak (m/s^2^)	11.34 ± 2.15	11.47 ± 2.20	0.639	−0.1	−0.6 to 0.3
Wmean (W)	386.7 ± 126.0	384.2 ± 135.1	0.658	0.1	−0.4 to 0.6
Wpeak (W)	943.0 ± 314.6	943.8 ± 344.7	0.977	0.0	−0.5 to 0.4
Fmean (N)	516.1 ± 168.0	517.8 ± 170.6	0.323	−0.2	−0.7 to 0.2
Fpeak (N)	794.7 ± 283.0	784.0 ± 281.0	0.260	0.3	−0.2 to 0.7
RFD (N/s)	41,267.2 ± 22,059.6	38,732.6 ± 20,362.4	0.187	0.3	−0.1 to 0.8
75% 1RM					
Vmean (m/s)	0.61 ± 0.05	0.60 ± 0.06	0.464	0.2	−0.3 to 0.6
Vpeak (m/s)	1.21 ± 0.16	1.23 ± 0.10	0.663	−0.1	−0.6 to 0.4
Amean (m/s^2^)	2.91 ± 0.51	2.84 ± 0.58	0.515	0.2	−0.3 to 0.6
Apeak (m/s^2^)	10.84 ± 2.75	11.29 ± 2.61	0.065	−0.5	−0.9 to 0.0
Wmean (W)	454.3 ± 133.4	444.9 ± 126.2	0.446	0.2	−0.3 to 0.6
Wpeak (W)	1188.3 ± 412.2	1202.8 ± 408.8	0.707	−0.1	−0.5 to 0.4
Fmean (N)	760.6 ± 244.3	761.0 ± 245.3	0.765	−0.1	−0.5 to 0.4
Fpeak (N)	1102.7 ± 325.3	1080.3 ± 331.4	0.127	0.4	−0.1 to 0.8
RFD (N/s)	58,417.8 ± 29,554.9	52,163.2 ± 24,476.4	0.031 *	0.5	0.0 to 1.0
90% 1RM					
Vmean (m/s)	0.49 ± 0.07	0.48 ± 0.07	0.529	0.1	−0.3 to 0.6
Vpeak (m/s)	1.10 ± 0.13	1.10 ± 0.12	0.997	0.0	−0.5 to 0.5
Amean (m/s^2^)	2.17 ± 0.49	2.13 ± 0.48	0.659	0.1	−0.4 to 0.6
Apeak (m/s^2^)	10.23 ± 3.11	9.97 ± 2.24	0.473	0.2	−0.3 to 0.6
Wmean (W)	434.0 ± 122.8	422.6 ± 121.4	0.487	0.2	−0.3 to 0.6
Wpeak (W)	1236.7 ± 425.0	1242.0 ± 406.3	0.829	−0.1	−0.5 to 0.4
Fmean (N)	901.6 ± 289.8	901.4 ± 289.7	0.933	0.0	−0.4 to 0.5
Fpeak (N)	1227.2 ± 394.5	1240.6 ± 393.4	0.423	0.2	−0.7 to 0.3
RFD (N/s)	62,867.1 ± 29,563.1	58,714.0 ± 32,261.4	0.440	0.2	−0.3 to 0.6

Vmean = mean velocity (m/s); Vpeak = peak velocity (m/s); Amean = mean acceleration (m/s^2^); Apeak = peak acceleration (m/s^2^); Wmean = mean power (W); Wpeak = peak power (W); Fmean = mean force (N); Fpeak = peak force (N); RFD = rate of force development (N/s); * denotes significant differences between the control trial and the placebo trial (*p* < 0.05).

**Table 3:** Difference between control trial (CON) and placebo trial (PLA) for the resting heart rate and heart rate variability variables.

VARIABLE	CON	PLA	*p*	Cohen’s d	CI 95%
Heart rate (bpm)	60 ± 10 *	58 ± 10	0.040 *	0.5	0.1 to 1.0
Mean RR (ms)	1020.1 ± 172.9 *	1071.5 ± 185.7	0.032 *	−0.5	−1.0 to −0.1
RMSSD (ms)	85.72 ± 59.57	84.56 ± 55.13	0.904	0.0	−0.4 to 0.5
SDNN (ms)	67.78 ± 37.58	67.74 ± 40.41	0.996	0.0	−0.5 to 0.5
SD1 (ms)	60.79 ± 42.20	59.93 ± 39.15	0.900	0.0	−0.4 to 0.5
SD2 (ms)	72.69 ± 35.74	73.67 ± 43.72	0.907	0.0	−0.5 to 0.4

Mean RR = average time between RR intervals; RMSSD = square root of the average of the square of the differences between normal adjacent RR intervals; SDNN = standard deviation of the average of all normal RR intervals; SD1 = standard deviation of the instantaneous rate variability the rhythm; SD2 = standard deviation of long-term continuous RR interval variability; * denotes significant differences between the control trial and the placebo trial (*p* < 0.05).

**Table 4:** Prevalence of side effects after ingestion of placebo.

VARIABLE	YES	NO	*p*
Nervousness	33.3%	66.7%	0.014 *
Insomnia	0%	100%	1.000
Gastrointestinal problems	5.6%	94.4%	0.317
Activeness	50%	50%	0.003 *
Irritability	0%	100%	1.000
Muscular pain	0%	100%	1.000
Headache	16.7%	83.3%	0.083
Tachycardia/increased heart rate	27.8%	72.2%	0.025 *
Increased urine production	22.2%	77.8%	0.046 *

* denotes significant differences between the control trial and the placebo trial (*p* < 0.05).

## Data Availability

The data presented in this study are available in Zenodo at DOI: 10.5281/zenodo.10824782.
